# The Review on Properties of Aloe Vera in Healing of Cutaneous Wounds

**DOI:** 10.1155/2015/714216

**Published:** 2015-05-19

**Authors:** Seyyed Abbas Hashemi, Seyyed Abdollah Madani, Saied Abediankenari

**Affiliations:** ^1^Department of Otolaryngology, Head and Neck Surgery, Traditional and Complementary Medicine Research Center, Mazandaran University of Medical Sciences, Sari, Iran; ^2^Traditional and Complementary Medicine Research Center, Mazandaran University of Medical Sciences, Sari, Iran; ^3^Immunogenetic Research Center, Faculty of Medicine, Mazandaran University of Medical Sciences, Sari, Iran

## Abstract

Treatment of wounds is very important and was subject of different investigations. In this regard, natural substance plays crucial role as complementary medicine. Various studies reported that aloe vera has useful effects on wounds especially cutaneous wounds healing. Therefore in the current review, we examined the effect of aloe vera on cutaneous wound healing and concluded that although aloe vera improves the wound healing as well as other procedures both clinically and experimentally, more studies are still needed to approve the outcomes.

## 1. Introduction

The skin plays an important role in protection from the body internal environment and it is the largest organ in human's body so exertion of serious damage to this organ may cause several problems in its survival. Skin is composed of two layers of epidermis and dermis that are placed over the subcutaneous adipose. Epidermis mainly comprises keratinocyte layers in which some other types of cells have spread including melanocytes and Langerhans cells. Epidermis has been separated from dermis by the basal membrane. Dermis is composed of papillary and reticular cells that comprise extracellular matrix or the basal substance consisting of collagen, fibrous networks, elastin, and glycosaminoglycans [1]. Despite various modern skin cares and treatments using herbal products like aloe vera plays an important role in wound healing especially in complementary medicine. Therefore in this paper we examined the effect of aloe vera on cutaneous wound healing.

## 2. Wound Healing, Classification, and Cell Signaling

Burning emerges as tissue trauma that is defined by some factors such as heat, chemicals, electricity, sunlight, and/or nuclear radiation. Most of burning cases are caused by building fires, touching the boiled water, water steam, liquids, and flammable gases [2].

The burning caused by heat and accidents and the like is assumed as the foremost cause of mortality and disability in the victims [3]. Two million people received medical treatments caused by burning traumas every year. Most of the primary treatments including drug topical dosage are employed to prevent against penetration of infectious substances into the wound. Improvement in methods of wound therapy and recovery of tissue may enhance the quality of life in the patients with burning trauma and also it may probably contribute to reducing the medical costs [4]. Treatment of dermal wounds is a complicated process that is the result of common performance between the various tissues with several cellular colonies. The behavior of each type of effective cells has been characterized during proliferation, migration, matrix building and contraction, phases and growth factors, and the existing matrix signals in the wound place at present [5]. A series of usual events may occur to treat the injury after trauma. As a result of inflammatory response due to trauma, the cells behind the dermis start building collagen and this continues up to the end of building epithelium [6].

Following incidence of any type of trauma on skin of other body tissues, a series of cellular and molecular events occurs. Wound healing includes three phases of thrombosis and inflammation, proliferation and formation of new tissue, and tissue retrieval [9]. The paramount cellular signaling events and extracellular matrix activities in healing process are controlled by various types of growth factors including fibroblastic growth factors (FGFs), epidermal growth factors (EGFs), transforming growth factors (TGFs), and insulin-like growth factors (IGFs). The insulin-like growth factor includes some peptides, which are very similar to insulin in terms of structural sequence and they are called somatomedin-C due to stimulation for secretion of growth factor hormone. These compounds play their role through various paths of autocrine, paracrine, and endocrine [10].

The insulin-like growth factors (IGFs) are secreted by few numbers of dermis and epidermis cells in normal skin but during dermal trauma they are secreted by most of epidermal cells including macrophages and platelets. This family of growth factors stimulates mitogenic fibroblasts and they also intervene in angiogenesis trend [11].

**Figure 1:**
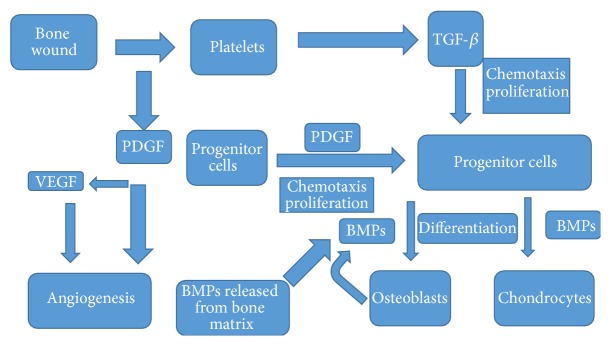
Signaling in wound healing: platelet-derived growth factor (PDGF), vascular endothelial growth factor (VEGF), and transforming growth factor-beta (TGF-*β*) [8].

Other studies have shown that IGFs along with other factors such as platelet-derived growth factors (PDGFs) play important role in process of wound healing so that they increase the thickness of dermis and epidermis. The rate of expression of IGF genes is low in epidermal basal layer, but this rate is significantly increased for one to three days after incidence of the wound [12, 13]. It has been shown in other investigations that abnormal increase in IGF may raise the expression and production of pro-alpha-I chain gene from collagen type-I and pro-alpha-I of collagen type-III in wound fibroblasts and as a result it will prepare the grounds for increase in size of scar (wound trace) [8, 13–16] (Figure 1).

Principally, the wound is defined as a lesion and rupture on skin surface that is caused by physical or thermal traumas, which need medical therapy. Improvement and healing of wound in human or developed animals occur with a completely complex and advanced mechanism as a result of passing through several phases including inflammation, proliferation, healing, and reconstruction [17]. Initially, the created gap on place of wound is closed quickly and epithelium is created again on surface of wound and a new matrix is substituted promptly with the lost skin. Nonetheless, if the above-said scheduled events are disrupted for any reason, the healing trend of skin wound encounters problem, and wound healing speed is affected by them. A lot of factors affect speed and quality of wound healing trend out of which one can refer to size of wound, blood storage on place, the existing external objects and microorganisms, age, health status, and nutrition state of patient [7, 18–20] (Table 1).

The other classification of wounds is based on quality and period of healing trend where accordingly the wounds can be divided into two groups of acute and chronic wounds. The acute wounds are called a group of injuries, which is usually surficial and they are healed completely within period of 8–12 weeks. The chronic wounds are said to be a group of wounds, which is slowly healed and period of their healing exceeds 12 weeks. These wounds are often recurring and continue. These wounds are created under conditions of special diseases like malignancies and chronic infections and/or under certain physiological conditions such as wounds in diabetic patients that occur in their body. The foot wounds with traumatic and ischemic and venal origin are considered as this group of wounds [21–24].

## 3. History

With respect to advancement of modern techniques for wound healing on the one hand and tendency to use healing property of medicinal herbs as a complementary medicine on the other hand during recent decades, this trend has caused creating of serious discussion and challenges about therapy of patients with dermal wounds and skin healing among the dermatologist physicians and specialists in plastic surgery [25, 26].

## 4. Pharmacognostical Characteristics

Aloe vera is a plant that belongs to Liliaceae family that grows easily in hot and arid regions. The existing mucilage tissue at the center of leaves in this plant that is also so-called aloe gel is used for various cosmetics and medical applications. The peripheral leaf cells in this plant produce bitter and yellow-color latex that is called aloes. Aloe vera is one of the plants, which can be noticed in this regard [27].

Aloe vera or yellow aloe is the herbaceous and perennial plant with thick, succulent, and long leaves. The margin of its leaves is a little curled with thistle. Its flowers are placed in beautiful clustering form at the end of florescent stem axis with green to yellow color. Aloe vera is endemic to African regions and it is also called desert lily (*Hesperocallis*) [28].

The Egyptians used aloe vera plant for treatment of wounds, burnings, and infections for the first time. After them, Greeks, Spanish, and African peoples used aloe vera plant by various techniques for several purposes. According to classic medicine in Iran, aloe vera has hot and dry humor and its extract is used for medicinal purpose [29, 30].

## 5. Chemical Constituents

Aloe vera plant is composed of anthracene hydroxyl derivatives including aloins A and B_2_ with total 25–40% of chromone compounds and derivatives such as aloe resins A, B2, and C. The other important compounds in aloe vera plant include several sugars such as glucose, mannose, and cellulose and various enzymes like oxidase, amylase, and catalase and also vitamins consisting of B_1_, B_2_, B_6_, C, E, and folic acid, and minerals like calcium, sodium, magnesium, zinc, copper, and chrome [31].

## 6. Therapeutic and Pharmacological Effects

One could refer to some of pharmacological activities attributed to aloe vera plant including anti-inflammatory, antiarthritis, antibacterial and antifungal, and hypoglycemic effects. Due to antibacterial and antifungal properties of aloe vera, this plant prevents against creation of dandruff on head. The aloe vera plant is also helpful for control of fungal infections such alopecia disease [32]. Of other effects, which have been ascribed to aloe vera fresh gel, one can imply its healing effects in wounds and skin surficial traumas. Similarly, reducing pain on place of trauma is seen following taking this drug [33]. The humid effects of aloe vera have been demonstrated in topical products of this plant as well [34]. The effects of aloe gel on skin improve the dermal intake of drugs as well. In a study that was carried out on effect of rising intake by aloe vera on drugs of caffeine, colchicines, mefenamic acid, oxybutynin, and kinin, this effect of skin rising intake was observed, which may be due to increase in water content (*stratum corneum*) [35]. Aloe vera (or yellow aloe) plant looks like cactus and a succulent and watery plant, whose leaves include mucilage tissue (gel). This mucilage consists of some glycoproteins, which prevent against inflation and pain and accelerate their improvement trend. Likewise, it comprises polysaccharides, which stimulate skin growth and healing. The mucilage of this plant can be used for treatment of internal and external wounds [36, 37].

## 7. Healing Mechanism

Its healing property is related to a compound that is called glucomannan, which is enriched with polysaccharides like mannose. The glucomannan affects fibroblast growth factor and stimulates the activity and proliferation of these cells and in turn improves collagen production and secretion. The mucilage of aloe vera not only increases amount of collagen on wound site, but also increases transversal connections among these bands rather than creation of change in collagen structure and as a result accelerates wound improvement [38]. Using medicinal herbs has been noticed in therapy of types of wound from the very beginning. Due to reduced financial load and its medical effects, these plants are noticed by the people. Several plants are used traditionally in treatment of many skin wounds and burnings in various points of the world [39]; among them one can refer to plants of jujube, mountain germander, olibanum, and also portulaca, whose effect has been proved during trend of healing of burning wounds in rats [40–42]. Inter alia, aloe vera is widely used as one of the plants with a very long history of healing of skin wounds and burnings.

It has been characterized that oral dosage of aloe vera mucilage by the rat with diabetes type-II has accelerated the trend of healing of skin wounds in these animals so that the results indicate that aloe vera treatment has accelerated the rising rate of expression in gene of vascular endothelial growth factor (VEGF) and TGF *β*-1 in the area of wound in skin of rats. In this case, TGF-*β*1 has stimulated fibroblasts to better reconstruct the extracellular matrix at wound place more than ever [43].

After creation of skin wound, the inflammatory responses and collagen rising production start by the cells in dermal area which is followed by rearrangement of epithelial tissue. This is a physiological process and many factors may intervene in it including growth factors and cytokines in quality of its trend. The wound healing is aimed at its therapy at the minimum possible time and with least amount of pain and ache and scar for the patient [44–46].

## 8. The Role of Growth Factors

The growth factors are proteins with heavy molecular weight that is produced by most of cells and as it secretes they start autocrine and paracrine autocascading mechanisms in various cellular processes. Among several effective growth factors in trend of wound healing one can imply transforming growth factors TGF-*β*. Following trauma in tissues, TGF-*β* is released from wound place by platelets degranulation [47].

In the study done by Roberts et al., upon occurrence of dermal trauma, TGF-*β*1 appears with platelet origin on the wound point and caused progress in wound healing trend [48].

TGF-*β*1 has increased mitosis power in fibroblasts of human's skin [49].

TGF-*β* causes increase in angiogenesis trend of various tissues by improving the expression of angiogenetic factors like VEGF in epithelial and fibroblast cells [50, 51]. TGF-*β* stimulates angiogenesis, proliferation of fibroblasts, differentiation of myofibroblasts, and formation of extracellular matrix [52].

TGF-*β*1 regulates and increases genetic expression of 2-FGF on site of skin wound [53].

In this course, other investigations have shown as well that *β*-sitosterol as one of the elements of aloe vera mucilage has increased angiogenesis and better healing of traumatic tissues by increase in rate of genetic expression of VEGF and its receptor on place of wound [54]. Also, experiments in vitro regarding stimulation of macrophages in the damaged tissues have shown that the existing mannose sugar in aloe vera compound stimulates these cells for producing cytokines and progress in some phases of wound healing after connection to the given receptor locating on surface of skin macrophages. In this sense, Trade Name of Acemenan has been assumed as a therapeutic compound for a group of polysaccharides enriched with mannose in aloe vera mucilage [55].

Atiba et al. (2011) showed that oral dosage of aloe vera mucilage might increase production of bFGF and TGF-*β*1 in place of wound on skin of rats, which had been exposed under a type of radiation [43]. Similarly, in another study (2010) it was identified that dermal dosage of aloe vera mucilage in wound place of rats has accelerated the healing as well as thrombosis and contraction of the wound point. It was known in this survey that dermal treatment with aloe vera has increased angiogenesis as well as granulated tissue and also better arrangement of collagen on wound site [56]. Topical dosage of aloe vera mucilage had led to healing of the shear wound in desert rat and rabbit [57].

## 9. The Role of Vitamins and Others

Aloe vera mucilage includes some compounds like vitamin E and vitamin C and some of amino acids, which can play important role in acceleration of wound healing trend in such a way that the experiments have indicated that vitamin C with increase in producing collagen and prevention from synthesis of these strands as well as vitamin E as a strong antioxidant enters in wound healing trend [58]. With antimicrobial and anti-inflammatory effects, aloe vera mucilage also causes the progress in wound healing trend [59]. The aloe vera mucilage possesses antioxidant enzymatic systems like glutathione peroxidase and superoxide dismutase, which accelerate wound healing trend by neutralization of effect of the free radicals produced on wound site and with their anti-inflammatory property [60].

A study by Daburkar et al. showed that use of aloe vera gel ethanolic extract attenuated the diabetic foot wound in rats [61]. Another article revealed that aloe vera could be a treatment of choice for burn injuries [62]. Oryan and coworkers [63] proved evidences that topical application of aloe vera would improve the biochemical, morphological, and biomechanical features of the healing cutaneous wounds in rats. A clinical trial investigation reported that aloe vera and* Calendula* ointment improve the speed of episiotomy wound healing; therefore it could be considered for quickening the episiotomy healing [64].

## 10. Conclusion

Considering aloe vera treatment for improvement of wound healing is useful as well as other standard treatments.

## Figures and Tables

**Table 1:** Growth factors in wound healing [7].

Growth factor	Source	Wound healing related functions
PDGF	Platelets, macrophages, endothelial cells, injured cells	Chemotaxis, fibroblast proliferation, collagenase production

TGF-*β*	Macrophages, platelets, neutrophils, lymphocytes, fibroblasts, epithelial and endothelial cells, injured cells	Chemotaxis, fibroblast proliferation, collagen metabolism

EGF	Macrophages, platelets, plasma, epithelial cells	Epithelial cells proliferation, granulation tissue formation

TGF-*α*	Activated macrophages, platelets, injured cells, epithelial cells	Epithelial cells proliferation, granulation tissue formation

KGF	Fibroblasts	Endothelial cells proliferation

IL-1	Macrophages	Fibroblast proliferation

FGF	Macrophages, fibroblasts, pituitary, endothelial cells	Fibroblast proliferation, matrix deposition, wound contraction angiogenesis

TNF-*α*	Macrophages, T lymphocytes	Fibroblast proliferation

IGF-1	Plasma, liver, fibroblasts	Synthesis of sulfated proteoglycans and collagen, fibroblast proliferation

IFNs	Lymphocyte, fibroblasts	Inhibition of fibroblast proliferation and collagen synthesis
